# A Comparative Study on Cu^2+^, Zn^2+^, Ni^2+^, Fe^3+^, and Cr^3+^ Metal Ions Removal from Industrial Wastewaters by Chitosan-Based Composite Cryogels

**DOI:** 10.3390/molecules25112664

**Published:** 2020-06-08

**Authors:** Doina Humelnicu, Ecaterina Stela Dragan, Maria Ignat, Maria Valentina Dinu

**Affiliations:** 1 Faculty of Chemistry, “Alexandru Ioan Cuza” University of Iasi, 700506 Iasi, Romania; doinah@uaic.ro (D.H.); maria.ignat@uaic.ro (M.I.); 2 Department of Functional Polymers, “Petru Poni” Institute of Macromolecular Chemistry, 700487 Iasi, Romania; sdragan@icmpp.ro; 3 Laboratory of Inorganic Polymers, “Petru Poni” Institute of Macromolecular Chemistry, 700487 Iasi, Romania

**Keywords:** chitosan, zeolite, cryogel sorbents, dynamic study, metal ions removal

## Abstract

Materials coming from renewable resources have drawn recently an increased attention in various applications as an eco-friendly alternative in the synthesis of novel functional materials. Polysaccharides, with their prominent representative – chitosan (CS), are well-known for their sorption properties, being able to remove metal ions from dilute solutions either by electrostatic interactions or chelation. In this context, we proposed here a comparative study on Cu^2+^, Zn^2+^, Ni^2+^, Fe^3+^, and Cr^3+^ metal ions removal from industrial wastewaters by CS-based composite cryogels using batch technique. The composite cryogels consisting of CS embedding a natural zeolite, namely clinoptilolite, were synthesized by cryogelation, and their sorption performance were compared to those of CS cryogels and of acid-activated zeolite. A deeper analysis of thermodynamics and kinetics sorption data was performed to get insights into the sorption mechanism of all metal ions onto sorbents. Based on the optimized sorption conditions, the removal of the above-mentioned ions from aqueous solutions by the composite sorbent using dynamic technique was also evaluated.

## 1. Introduction

Industrial activities, such as mining operations, manufacturing of electronic devices, electroplating, chemical etching, and petro–chemistry, are mainly responsible for the release of heavy metal ions (HMIs) into the environment, which represents a serious threat to the human health, living entities and all the ecological systems. A broad variety of HMIs, including copper, nickel, cadmium, cobalt, zinc, iron, lead, or chromium can be detected into the industrial wastewaters, whose concentrations are much higher than those recommended by the environmental agencies.

Coagulation/flocculation, membrane filtration, electrochemical processes, electrodialysis, ion exchange, photocatalysis, and biological treatments [1,2] are some of the wastewater decontamination methods in continuous development for diminishing the harmful effect of the HMIs onto the ecosystem. However, these techniques have several major limitations, e.g., an incomplete metal ion removal, request of extra chemicals, production of large amounts of sludge, high operating costs, and so forth. To overcome these shortcomings, many studies have been focused on adsorption processes [1], due to its sustainability, low fouling problems, high efficiency, low energy consumption, easy operation, as well as less secondary pollution.

Various sorbents including activated carbon [3], carbon nanotubes [4], organic ion exchangers [5], zeolites [6,7,8,9], and polysaccharide-based hydrogels [10,11,12,13,14,15,16] have been tested for the treatment of HMIs-containing wastewaters. Among them, polysaccharides, such as chitosan (CS) [10,11,12], salecan [13], pullulan [14], alginate [15], and pectin [16], as biosorbents, are preferred because they possess several advantages such as high abundance, biodegradability, and nontoxicity, particularly suited for the removal of HMIs from polluted tap or groundwater. Among the available biopolymers, CS has the highest sorption performance toward HMIs [10,11,12] due to its abundant -NH_2_ and -OH groups. Its sorption capacity has been further improved by functionalization with diacetylmonoxime [17] and polyhexamethyleneguanidine [18] or grafting of malic acid [19] or glutamic acid [20] onto CS matrix. Nevertheless, CS has poor mechanical properties and exhibits low chemical stability in harsh aqueous environments such as HMIs-containing wastewaters. Consequently, crosslinking [21], blending with other natural or synthetic polymers [22,23,24,25], imprinting [26,27], or incorporating of reinforcing fillers such as magnetite [28,29], zeolites [22,30,31,32], or metal-organic frameworks [33] into the CS matrices have been explored to design composite materials endowed with enhanced sorption capacity, selectivity, and reusability performance, especially toward Cu^2+^ ions using simulated aqueous solutions. However, the real wastewaters consist of complex mixtures of HMIs in various concentrations. This highlights the necessity to evaluate the low-cost CS-based sorbents to remove Cu^2+^ ions from multicomponent mixtures with a much broader variety of HMIs.

The objectives of this study are (i) to use a composite based on clinoptilolite (CPL) particles entrapped within CS cryogels for the treatment of high-concentrated wastewaters containing Cu^2+^, Zn^2+^, Ni^2+^, Fe^3+^, and Cr^3+^ ions resulted from photoetching processes; (ii) to systematically evaluate the effects of several parameters such as the solution pH, sorbent dose, number of sorption/desorption cycles, contact time, and temperature on the sorption performance of CS-based composite cryogel in comparison to that of cross-linked CS cryogel and of acid-treated clinoptilolite (CPLH^+^) sorbents; (iii) to explain the sorption mechanism by fitting non–linear theoretical models onto experimental sorption kinetics data; (iv) to evaluate the sorption performance of CS-based composite cryogel in dynamic continuous mode.

## 2. Results and Discussion

**Figure 1:**
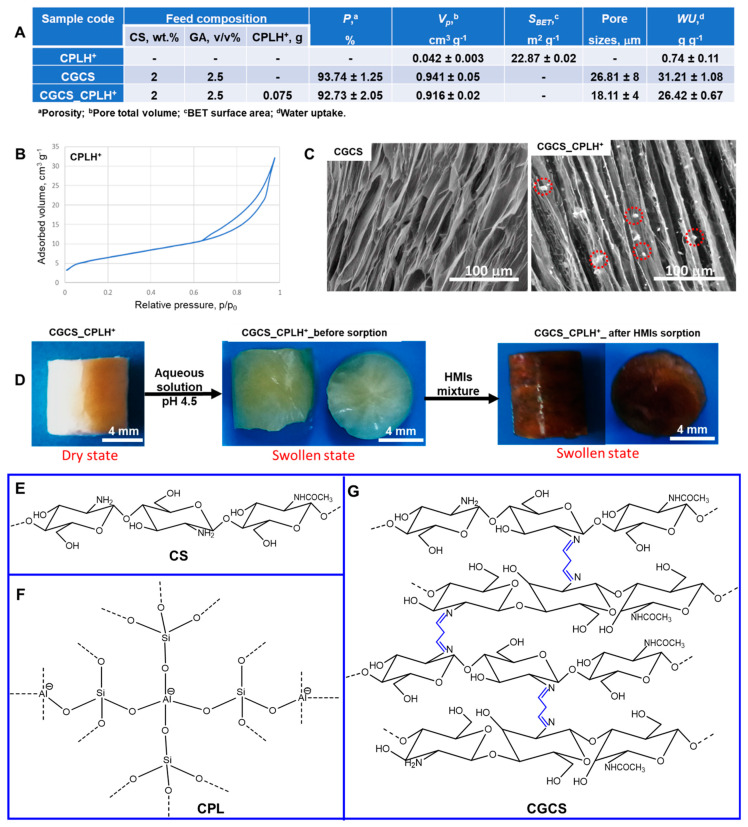
(**A**) Some characteristics of the sorbents; (**B**) N_2_ adsorption–desorption isotherm on CPLH^+^ sorbent; (**C**) SEM micrographs of CS–based sorbents; the red circles on the SEM micrograph of CGCS_CPLH+ composite cryogel represent CPLH^+^ particles; (**D**) Optical pictures of CGCS_CPLH^+^ composite cryogels before and after HMIs loading; (**E**) Chemical structure of CS; (**F**) Tetrahedral representation of CPL which consists of [AlO_4_]^5−^ and [SiO_4_]^4−^ units linked together by oxygen bridges; (**G**) A schematic diagram showing the structure of CGCS sorbent (in blue color is marked the cross–linking with GA).

Compared to powdery or flake CS, CS-based cryogels prepared by unidirectional freezing methodology have attractive advantages, including high porosity and large pore sizes (Figure 1), which may facilitate a fast diffusion of HMIs toward a large number of –NH_2_ and−OH functional groups of the sorbent (Figure 1G). The stable monolith shape of the CS-based sorbent (Figure 1D) ensures its easy separation. Therefore, cross-linked CS cryogels and CGCS_CPLH^+^ composite cryogels were tested as sorbents for Cu^2+^, Zn^2+^, Ni^2+^, Fe^3+^, and Cr^3+^ ions removal under competitive conditions. Their sorption performance as a function of several parameters were systematically investigated in comparison to CPLH^+^ sorbent. Some characteristics of the sorbents used in this study were collected in Figure 1.

The nitrogen adsorption-desorption BET isotherm of CPLH^+^ is categorized as a Type IV isotherm (Figure 1B), indicating the presence of mesopores [34]. The BET surface area and the pore volume of the CPH^+^ particles were determined as 24 m^2^·g^−1^, and 0.042 cm^3^·g^−1^ (Figure 1A). The CS-based sorbents have a well-patterned morphology (Figure 1C) with porous channels unidirectionally arranged with an average distance between them of about 27 µm for CGCS and of about 18 µm for CGCS_CPLH^+^. The presence of CPLH^+^ particles on the surface of CGCS_CPLH^+^ composite cryogel could be observed on the SEM micrograph (Figure 1C – red circles on the right SEM micrograph). The optical images in Figure 1D shows the monolith shape of the CGCS_CPLH^+^ composite sorbent, which is preserved after loading with HMIs. The CGCS_CPLH^+^ composite sorbent was colored in dark brown after sorption of HMIs from industrial wastewater (Figure 1D).

### 2.1. Sorption Properties of Cryogel-Based Sorbents in Bach Mode

#### 2.1.1. Removal of Divalent Cations (Cu^2+^, Zn^2+^, and Ni^2+^) under Competitive Conditions

**Figure 2:**
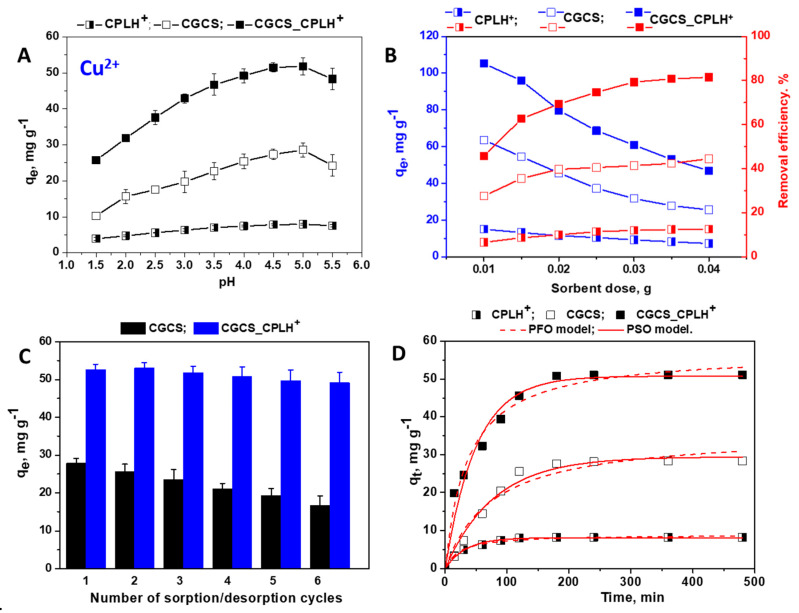
Influence of (**A**) pH values, (**B**) sorbent dose, (**C**) successive sorption/desorption cycles, and (**D**) contact time onto Cu^2+^ ions removal from multicomponent mixtures by CPLH^+^, CGCS, and CGCS_CPLH^+^ sorbents.

Cu^2+^, Zn^2+^, and Ni^2+^ ions were investigated for their affinity to the CGCS and CGCS_CPLH^+^ cryogel matrices in comparison to that of CPLH^+^ sorbent. Batch-mode sorption studies were performed under competitive conditions using five-component industrial wastewater. The concentration of each cation in the five–component wastewater was 230 mg·L^−1^ for Cu^2+^ ions, 63 mg·L^−1^ for Zn^2+^, 230 mg·L^−1^ for Ni^2+^, 230 mg·L^−1^ for Fe^3+^ ions, and 6 mg·L^−1^ for Cr^3+^ ions, respectively. The sorption properties of Cu^2+^ ions from this HMIs mixture, as a function of pH, sorbent dose, number of sorption/desorption cycles, and contact time are presented in Figure 2.

As Figure 2A shows, the amount of Cu^2+^ ions retained by CS–based cryogel sorbents (CGCS, and CGCS_CPLH^+^) significantly increased with the increase of pH from 1.5 to 5, and decreased afterwards. In the case of CPLH^+^ sorbent, only a slight increase of the *q_e_* values was observed in the studied pH range. At pH > 5, the precipitation of Cu(OH)_2_ occurs, and thus the *q_e_* values started to decrease. The influence of pH on the removal of Cu^2+^ ions by the CS-based cryogels is almost similar with that reported for other sorbents containing CS as matrix [19,20,23,24,29,30,35,36]. Based on the above results, the pH value of the wastewater was adjusted to 5 when the sorption of Cu^2+^ ions was further evaluated.

The sorbent dosage is also an important parameter for removal of Cu^2+^ ions [14,23,36]. Consequently, the Cu^2+^ removal from its mixture with Zn^2+^, Ni^2+^, Fe^3+^ and Cr^3+^ ions by CPLH^+^, CGCS, and CGCS_CPLH^+^ sorbents with different dosages was investigated. As presented in Figure 2B, we found that the amount Cu^2+^ retained (*q_e_*, mg·g^−1^) by sorbents decreased with the increase of sorbent dosage, while the removal efficiency (RE, %) increased with the increase of sorbent dosage. This behavior is associated with many sites/functional groups able to remove efficiently a high quantity of HMIs, while the number of HMIs distributed per sorbent mass unit decreased, accordingly, with the increase of the sorbent dose [14,23,36]. For instance, the saturated sorption capacity of CPLH^+^, CGCS, and CGCS_CPLH^+^ sorbents decreased from 15.01 mg·g^−1^, 63.54 mg·g^−1^ and respectively, 105.18 mg·g^−1^ to 7.23 mg·g^−1^, 25.54 mg·g^−1^, and respectively, 46.86 mg·g^−1^ (Figure 2B) when the sorbent dosage increased from 0.01 to 0.04 g. The RE values were around 12%, 40%, and 80% for CPLH^+^, CGCS, and respectively, CGCS_CPLH^+^ sorbents at a sorbent dose higher than 0.03 g. A RE value higher than 80% was achieved when the Cu^2+^ ions removal was studied using single-component aqueous systems [14,20,35,36]. Considering the RE values, the optimal sorbent dose of 0.035 g was chosen for all upcoming sorption experiments.

In this study, the HMIs retained on CS-based sorbents were successfully eluted with 0.1 M HCl solution. The regeneration in convenient conditions, the level of reusability, and the recovery of HMIs in a concentrated form are key factors, which should be taken under consideration when the feasibility of a sorbent is evaluated [23,35,36]. In this respect, the CSCS and CGCS_CPLH^+^ sorbents were further involved in successive sorption/desorption cycles (Figure 2C), after the adequate regeneration with 0.1 M NaOH. For the CGCS sorbent, a slight decrease of the *q_e_* values was observed after the 3^rd^ sorption/desorption cycle. As can be seen from Figure 2C for the CGCS_CPLH^+^ sorbent, the *q_e_* values remained almost unchanged even after the 5^th^ cycle of sorption/desorption, revealing its remarkable chemical stability under harsh conditions.

The effect of contact time on the sorption of Cu^2+^ onto the CPLH^+^, CGCS, and CGCS_CPLH^+^ sorbents was studied, and as it can be seen (Figure 2D) the sorption capacity shows a sharp increase from the beginning due to a high number of available active sites. Then, as the active sites are filled up there is a slow increase till the equilibrium state [24]. The optimum contact time was set at 180 min.

**Figure 3:**
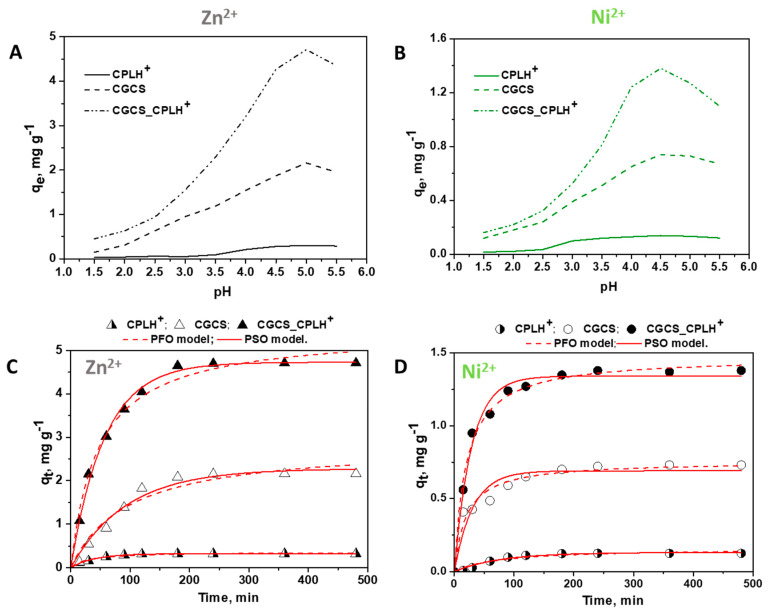
Influence of pH values (**A**,**B**) and contact time (**C**,**D**) onto Zn^2+^ (**A**,**C**) and Ni^2+^ (**B**,**D**) ions removal from multicomponent mixtures by CPLH^+^, CGCS, and CGCS_CPLH^+^ sorbents.

The removal of the other two competitive divalent cations, i.e., Zn^2+^ and Ni^2+^ ions by CPLH^+^, CGCS and CGCS_CPLH^+^ sorbents was also first investigated as a function of pH value of the multi-component industrial wastewater (Figure 3A,B).

As Figure 3 shows, at pH lower than 4, both divalent ions (Zn^2+^ and Ni^2+^) were less retained on the sorbents. The higher sorption capacity was obtained at pH 5 for Zn^2+^ ions and at pH 4.5 for Ni^2+^ ions. Further increase in pH leads to the precipitation of Zn^2+^ or Ni^2+^ hydroxide complexes which inhibits the sorption process. Compared to Cu^2+^ ions, the CPLH^+^, CGCS, and CGCS_CPLH^+^ sorbents showed a low affinity for the Zn^2+^ and Ni^2+^ ions (Figure 2 and Figure 3). In conclusion, the studies on the pH effect on the HMIs removal by CPLH^+^, CGCS, and CGCS_CPLH^+^ sorbents showed that the q_e_ values increase with the increase of pH, up to an optimum pH for each metal ion, and was reduced thereafter. The explanation for this behavior is based on the ionic speciation in solution as a function of pH [35]. At pH values lower than 4, the competition between H^+^ and Cu^2+^, Zn^2+^, and Ni^2+^ ions for the sorption sites of the sorbents significantly diminished the sorption of HMIs on the surface of the CPLH^+^, CGCS, and CGCS_CPLH^+^ sorbents. In addition, at low pH the CS-based sorbents are positively charged, the protonation of -NH_2_ groups in acidic solution induces an electrostatic repulsion of HMIs that reduces the number of binding sites available for HMIs [20,23,29,37,38]. However, the Cu^2+^, Zn^2+^ and Ni^2+^ ions uptake increased as the pH increased to pH 5.0 and respectively to 4.5 (Figure 2 and Figure 3), as most active sites on the CS-based sorbents are deprotonated [23,35] resulting in a more net attractive force which is responsible for high HMIs removal from aqueous solution. In the case of CPLH^+^ sorbent, it was showed that the sorption of divalent cations (Cu^2+^, Ni^2+^ or Zn^2+^ ions) by ion exchange was also enhanced by increasing the initial pH of the solution. At acidic pH values, ion exchange sorption is reduced due to the competition of these ions and H^+^ ions for dynamic ion exchange sorption sites [7,9]. With decreasing H^+^ ions in the solution at higher pH values, the zeolite surface is deprotonated and subsequently the uptake of the metal ions is enhanced [7,9].

To understand the influence of contact duration on the sorption properties of HMIs on all sorbents, a series of experiments was performed at various times between 15 and 480 min (Figure 3C,D). The greatest rate of sorption was exhibited by CPLH^+^ sorbent and occurred within about 50 min for Zn^2+^ ions and 90 min for Ni^2+^ ions. This rapid sorption can be attributed to HMIs sorption by an ion exchange reaction on the surface sites rather than in the pores [7]. The sorption mechanism is associated with the rate-limiting step in a sorption process [23,24,35]. To get insights into the process dynamics and to determine the sorption rate of HMIs onto sorbents, pseudo-first order model (PFO) and pseudo-second order model (PSO) (Table 1) were employed.

The experimental q_e_ values for Cu^2+^ ions removal by CPLH^+^, CGCS, and CGCS_CPLH^+^ sorbents were 8.2 mg·g^−1^, 28.29 mg·g^−1^, and respectively, 51.15 mg·g^−1^ (Figure 2D). The experimental q_e_ values for Zn^2+^ ions removal by CPLH^+^, CGCS, and CGCS_CPLH^+^ sorbents were 0.32 mg·g^−1^, 2.16 mg·g^−1^, and respectively, 4.71 mg·g^−1^ (Figure 3C). The experimental *q_e_* values for Ni^2+^ ions removal by CPLH^+^, CGCS, and CGCS_CPLH^+^ sorbents were 0.12 mg·g^−1^, 0.73 mg·g^−1^, and respectively, 1.38 mg·g^−1^ (Figure 3D). The high values of R^2^, the low values of χ^2^, and the negligible differences between the calculated (*q_e,calc_*) and the experimental capacities suggest that the PSO model is the predominant kinetic model for Cu^2+^, Zn^2+^, and Ni^2+^ ions removal by all sorbents (Figure 2D, Figure 3C,D, and Table 1). The obtained results are in agreement with the literature data regarding the sorption kinetic analysis of Cu^2+^, Zn^2+^, Ni^2+^ ions onto other CPL [6,7,8,9] or CS-based sorbents [18,20,23,24,36,39,40,41], which have been well fitted with PSO kinetic. According to a PSO model assumption the rate–determining step may be chemisorption, relating valence forces through sharing or exchange of electrons between HMIs and active sites [23,24].

#### 2.1.2. Removal of Trivalent Cations (Fe^3+^ and Cr^3+^) under Competitive Conditions

**Figure 4:**
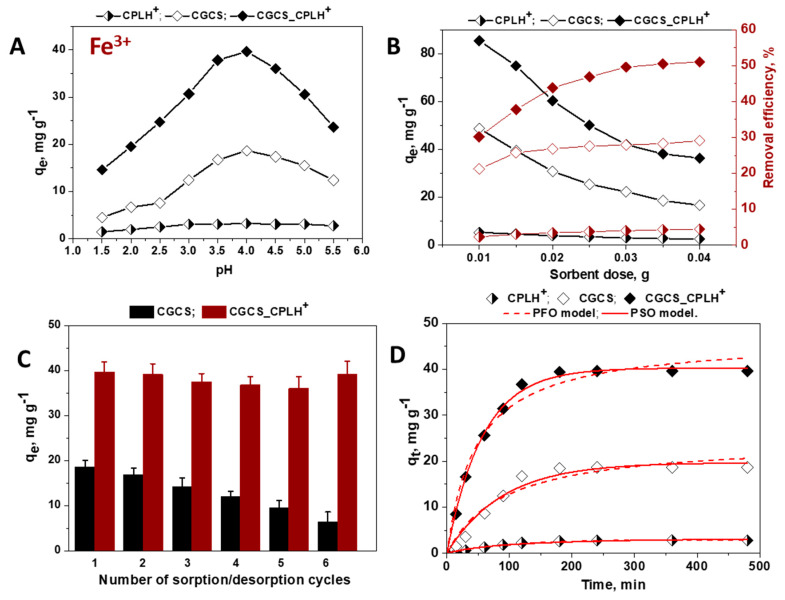
Influence of pH values (**A**), sorbent dose (**B**), successive sorption/desorption cycles (**C**), and contact time (**D**) onto Fe^3+^ ions removal from multicomponent mixtures by CPLH^+^, CGCS, and CGCS_CPLH^+^ sorbents.

The effect of pH, sorbent dose, number of sorption/desorption cycles, and contact time was also first systematically investigated for removal of Fe^3+^ ions from multicomponent industrial wastewaters using CPLH^+^, CSCG, and CSCG_CPLH^+^ sorbents (Figure 4).

As Figure 4A shows the sorption of Fe^3+^ increased with increasing pH of the solution, the optimum sorption pH being around 4.0. In the solution pH range 1 to 5, Fe^3+^ appeared as Fe^3+^, FeOH^2+^, Fe(OH)_2_^+^, Fe_2_(OH)_2_^4+^, and Fe_3_(OH)_4_^5+^ cationic species [35]. The presence of Fe(OH)^2+^ as major species at pH > 4, induced the decrease in the sorption capacity for these metal ions (Figure 4A). A similar behavior has been reported for the sorption of Fe^3+^ on other CS-based sorbents [35,42].

The increase of the sorbent dose (Figure 4B) led to the decrease of the *q_e_* values and to the increase of the RE values, as expected. For example, the saturated sorption capacity of CPLH^+^, CGCS, and CGCS_CPLH^+^ sorbents decreased from 5.38 mg·g^−1^, 48.78 mg·g^−1^ and respectively, 69.43 mg·g^−1^ to 2.58 mg·g^−1^, 16.74 mg·g^−1^, and respectively, 29.35 mg·g^−1^ when the sorbent dosage increased from 0.01 to 0.04 g. The RE values were around 4%, 29%, and 50% for CPLH^+^, CGCS, and respectively, CGCS_CPLH^+^ sorbents when the sorbent dose ranged from 0.03 g to 0.04 g (Figure 4B).

The Fe^3+^ ions retained on all sorbents were successfully desorbed with 0.1 M HCl solution, and after the regeneration with 0.1 M NaOH solution the CS-based sorbents were reused in six consecutive sorption/desorption cycles (Figure 4C). In the case of CGCS sorbent, the sorption capacity decreased after each sorption/desorption step indicating its low chemical stability. However, the CGCS_CPLH^+^ sorbent exhibited a remarkable chemical stability during the loading/leaching steps of Fe^3+^ ions, the sorption capacity remaining almost constant even after the 6*^th^* cycle.

The effect of the contact time on the Fe^3+^ ions retention capacity of the sorbents is shown in Figure 4D. The time necessary to reach sorption at equilibrium of Fe^3+^ ions from five-component aqueous solution was about 50 min for CPLH^+^, and about 120 min for CGCS and CGCS_CPLH^+^ sorbents (Figure 4D).

**Figure 5:**
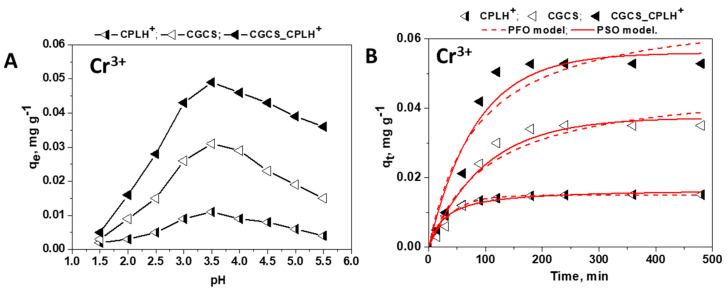
Influence of (**A**) pH values and (**B**) contact time onto Cr^3+^ ions removal from multicomponent mixtures by CPLH^+^, CGCS, and CGCS_CPLH^+^ sorbents.

The sorption of Cr^3+^ ions onto CPLH^+^, CGCS and CGCS_CPLH^+^ sorbents was also strongly affected by the pH of the solution (Figure 5A).

As it was already observed for other HMIs, the sorption capacity of Cr^3+^ ions increased also with the increase of pH, the optimum sorption pH being located at 3.5 (Figure 5A). Besides the explanation included above for divalent cations, in the case of Cr^3+^ ions the results could be also associated with the speciation of Cr^3+^ ions in solutions. Thus, Cr^3+^ has been reported to exist as Cr^3+^, CrOH^2+^, Cr_2_(OH)_2_^4+^ cationic species when the solution pH ranged from 1 to 5 [35]. The presence of Cr(OH)^2+^ as major species at pH > 3.5, induced the decrease in the sorption capacity for these metal ions (Figure 5A). An optimum sorption pH of 3.8 for Cr^3+^ ions has been also reported for CS flakes [12].

The effect of the contact time on the Cr^3+^ retention capacity of the CPLH^+^, CGCS, and CGCS_CPLH^+^ sorbents is presented in Figure 5B. The contact time varied in the range 0–480 min. As Figure 5B shows, the time required to achieve the equilibrium at pH 3.5 was about 120 min for all sorbents.

The sorption kinetics data of Fe^3+^ and Cr^3+^ ions onto CPLH^+^, CGCS, and CSCG_CPLH^+^ sorbents were also fitted by PFO and PSO models (Figure 4D and Figure 5B), and the obtained kinetic parameters are presented in Table 2.

The experimental q_e_ values for Fe^3+^ ions removal by CPLH^+^, CGCS, and CGCS_CPLH^+^ sorbents were 2.81 mg g^−1^, 18.64 mg g^−1^, and respectively, 39.64 mg g^−1^ (Figure 4D). The experimental q_e_ values for Cr^3+^ ions removal by CPLH^+^, CGCS, and CGCS_CPLH^+^ sorbents were 0.015 mg·g^−1^, 0.035 mg·g^−1^, and respectively, 0.053 mg·g^−1^ (Figure 5B). As can be seen in Table 2, the kinetic data for the sorption of Fe^3+^ and Cr^3+^ ions onto all sorbents under study were well described by the PSO kinetic model, the values of R^2^ and of the χ^2^ being higher and respectively lower for these models than for the PFO kinetic model. These results support also a chemisorption mechanism for Fe^3+^ and Cr^3+^ ions sorption.

For a comparative evaluation, the sorption properties of CPLH^+^, CGCS, and CGCS_CPLH^+^ sorbents toward Cu^2+^, Zn^2+^, Ni^2+^, Fe^3+^ and Cr^3+^ ions were also investigated by carrying out equilibrium sorption experiments in single-component system (Figure S1). Langmuir, Freundlich, Sips, Temkin and Dubinin–Radushkevich (D–R) isotherm models [24,41,42,43] were further used to investigate the sorption at equilibrium of the systems and to define the maximum sorption capacity (*q_m_*, mg g^−1^) of CPLH^+^, CGCS, and CGCS_CPLH^+^ sorbents (Figure S1). The Langmuir isotherm model fitted the experimental sorption equilibrium data better than Freundlich isotherm model, revealing homogenous distribution of the chelating sites on the sorbents for a uniform interaction with HMIs (Tables S1–S3). The separation factor, R_L_, values revealed a favorably sorption process, and the higher values of Freundlich constant, K_F_, obtained for CGCS_CPLH^+^, irrespective of the investigated HMI, supported the capacity enhancement by addition of CPLH^+^ onto CGCS cryogel network. Parameter n indicated the strong sorption of Cu^2+^ and Fe^3+^ ions onto sorbents as it lies in the range 2–4 (Tables S1–S3), indicating chemisorption as the main mechanism of sorption process [24,44]. The values of the mean free energy of sorption, E (kJ mol^−1^), evaluated taking into account the D–R isotherm constant (Equation (10), Supporting Information) of Cu^2+^, Zn^2+^, Ni^2+^, Fe^3+^, and Cr^3+^ ions onto CPLH^+^ sorbent (Table S1) were ranging from 12.5 to 15.15 kJ mol^−1^, indicating a process which occurs by an ion exchange mechanism. In the case of CGCS_CPLH^+^ sorbent the values of E for removal of Cu^2+^ and Fe^3+^ ions were higher than 40 kJ mol^−1^, supporting a process which occurred by chemisorption (Table S3). The q_DR_ values were in the same range as the values of q_m_ obtained by fitting the Langmuir isotherm (Tables S1–S3), which supports the applicability of the D−R isotherm in describing the sorption process. Subsequently, the compatibility of the Sips isotherm model for all sorbents, sustained by the high values of R^2^, as well as the calculated values of q_m_, which were very close to the experimental ones, points out the homogeneous distribution of the active sites/functional groups onto the sorbent surface. The maximum theoretical sorption capacities of CSCS_CPLH^+^ composite sorbent according to Sips model toward Cu^2+^, Zn^2+^, Ni^2+^, Fe^3+^, and Cr^3+^ ions were 61.10 mg·g^−1^, 18.67 mg·g^−1^, 12.24 mg·g^−1^, 53.46 mg·g^−1^, and respectively, 0.85 mg·g^−1^ (Tables S1–S3). Table S4 includes the maximum sorption capacity of various sorbents for Cu^2+^, Zn^2+^, Ni^2+^, Fe^3+^, and Cr^3+^ ions removal in comparison to that of our investigated sorbents. NaCPL [6,8], MnO_2_-coated zeolite [7], aluminosilicates modified by *N*,*N*’-bis(3-triethoxysilylpropyl)thiocarbamide [45], CS flakes [12], cross-linked CS gels [42], glutamic-CS hydrogels [20], CS/starches-g-PAN cryobeads [23], or CS/poly(vinyl amine) composite beads [38] have been successfully employed for removal of Cu^2+^, Zn^2+^, Ni^2+^, Fe^3+^, or Cr^3+^ ions from single-component aqueous solutions (Table S4). It should be pointed out that their sorption properties depended on the initial metal ion concentration, pH, sorbent dose, presence of other competitive species or temperature at which the sorption was carried out.

#### 2.1.3. Thermodynamic Studies

The temperature effect on the sorption of Cu^2+^, Zn^2+^, Ni^2+^, Fe^3+^, and Cr^3+^ ions from industrial wastewater onto CPLH^+^, CGCS, and CGCS_CPLH^+^ sorbents was studied at 293, 298, 303, 308, and 313 K for 6 h of contact time at 125 rpm. The results showed that the sorption capacity of all metal ions increased with temperature increment, indicating an endothermic in nature process. The distribution coefficient K_D_ (L mg^−1^) was calculated by equation [35,43,46]:

(1) $$K_{D}=\frac{q_{e}}{C_{e}}$$

Gibbs free energy change (ΔG^0^, kJ mol^−1^), enthalpy change (ΔH^0^, J mol^−1^) and entropy change (ΔS^0^, kJ mol^−1^ K^−1^) were calculated using the following equations [35,43,46]:

(2) $$\Delta G^{o}=-RTlnK^{o}\;\mathrm{and}\;K^{o}=K_{D}\times M_{adsorbate}\times 55.5$$

(3) $$lnK^{o}=\frac{\Delta S^{o}}{R}-\frac{\Delta H^{o}}{RT}$$

**Figure 6:**
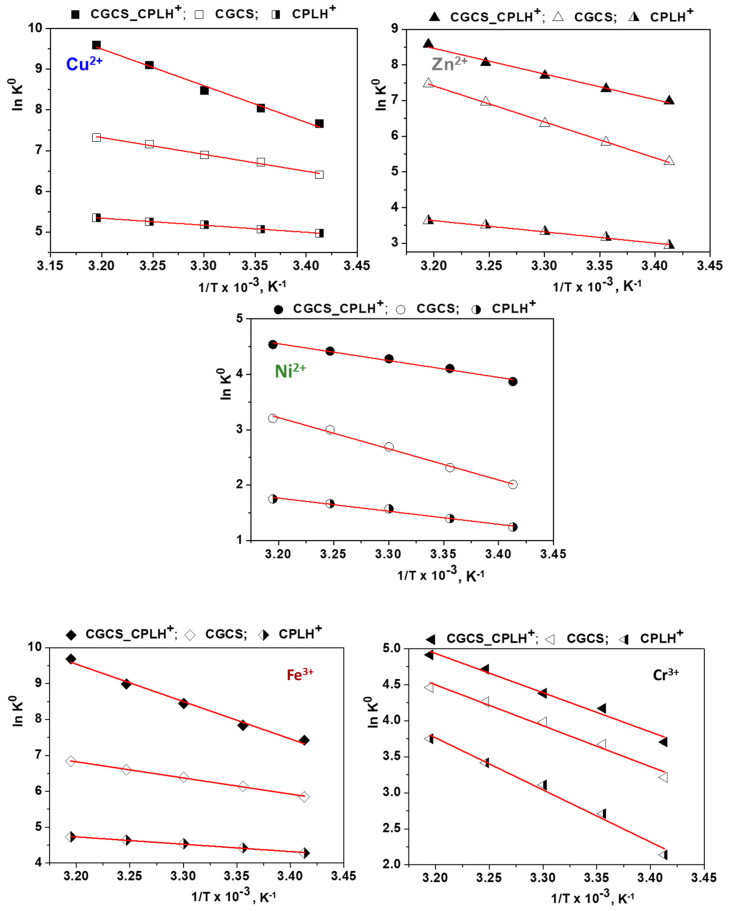
Plot of *lnK^o^* versus *1/T* for the sorption of Cu^2+^, Zn^2+^, Ni^2+^, Fe^3+^, and Cr^3+^ ions onto CS-based sorbents.

The values of ΔH° and ΔS° were calculated from the slope and intercept of the plot ln*K^0^* vs 1/T, respectively (Figure 6, Table 3).

The negative values of ΔG° reveal the spontaneous nature and feasibility of the sorption process of Cu^2+^, Zn^2+^, Ni^2+^, Fe^3+^, and Cr^3+^ ions onto CPLH^+^, CGCS, and CGCS_CPLH^+^ sorbents. The higher the negative values, the more the favorable conditions for the ion sorption [24,35,47]. The positive ΔH° values confirmed that the process is endothermic in nature. The positive ΔS° values indicate increased randomness at the solid-solution interface during the sorption processes [24,35,47]. The adsorbate species displace the adsorbed water molecules and the latter gain more translational entropy than is lost by the former, resulting in prevalence of randomness in the system [24,35,46,48].

### 2.2. Sorption Properties of Cryogel-Based Sorbents in Dynamic Mode

**Figure 7:**
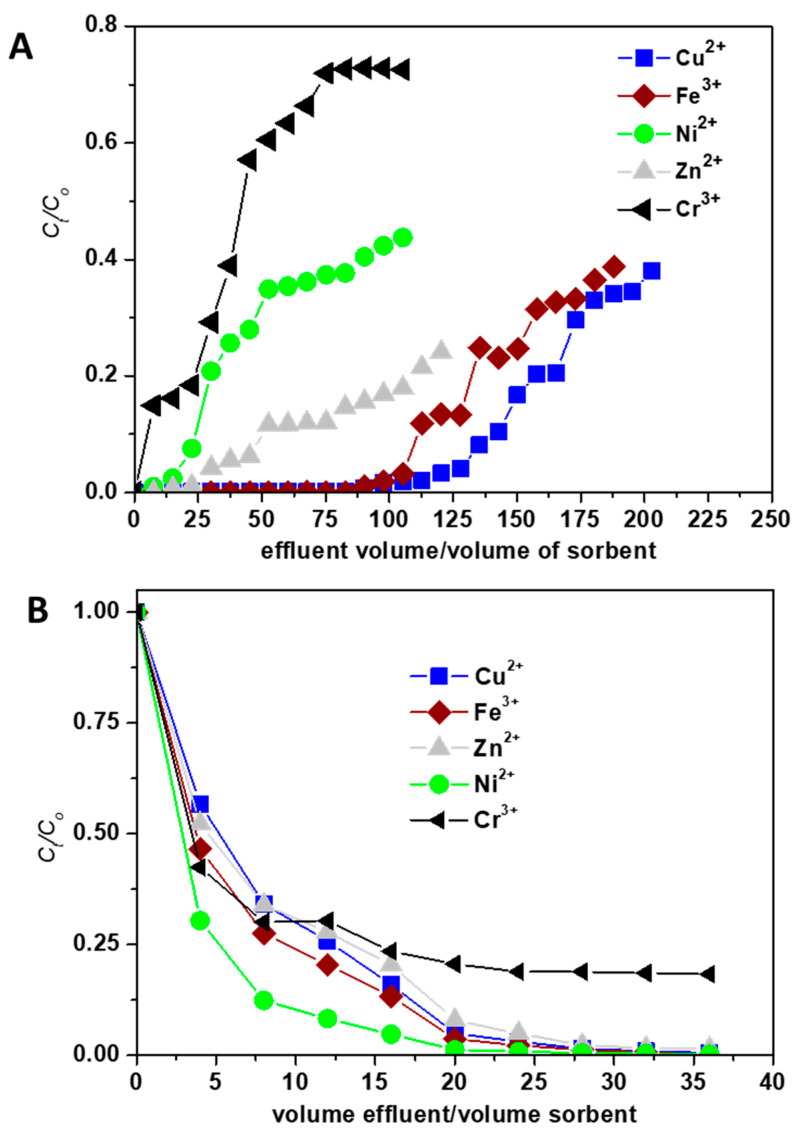
Sorption (**A**) and desorption (**B**) performance of CGCS_CPLH^+^ composite sorbent in treating industrial wastewaters containing Cu^2+^, Zn^2+^, Ni^2+^, Fe^3+^, and Cr^3+^ ions under fixed-bed column conditions.

The batch sorption experiments showed a great potential of the CGCS_CPLH^+^ composite sorbent in treating industrial wastewaters containing Cu^2+^, Zn^2+^, Ni^2+^, Fe^3+^, and Cr^3+^ ions. Therefore, its affinity against HMIs was preliminary evaluated in dynamic conditions. The obtained data are presented in Figure 7. The CGCS_CPLH^+^ composite sorbent exhibited a high selectivity toward Cu^2+^ ions, this HMI appeared in the effluent after ca. 110 bed volumes (Figure 7A).

The Cr^3+^ and Ni^2+^ ions have relatively low affinity toward the CGCS_CPLH^+^ composite sorbent and the breakthrough was situated at ca. 7.5 and 25 bed volumes for Cr^3+^ and Ni^2+^, respectively. Loading curves (Figure 7A) followed the same order as in the case of batch experiments under competitive conditions: Cu^2+^ > Fe^3+^ > Zn^2+^ > Ni^2+^ > Cr^3+^. The desorption of HMIs retained onto composite sorbent during fixed-bed column experiments was performed using 0.1 M HCl. As can be seen in Figure 7B, Cu^2+^, Fe^3+^, Zn^2+^, Ni^2+^ were completely desorbed at 30 bed volumes, while the Cr^3+^ ions recovery was only about 75%. As the sorption of the HMIs onto sorbents under dynamic conditions are more complex and are influenced by the bed height, feed flow rate, or initial HMIs concentration, future experiments will be dedicated to the investigation of the influence of these parameters onto HMIs removal from multicomponent systems.

## 3. Materials and Methods

### 3.1. Materials

Chitosan (CS), as powder, was purchased from Sigma-Aldrich Chemie GmbH (Schnelldorf, Germany). The average molecular weight and the deacetylation degree of CS were determined as 330 kDa and respectively, 85%. The clinoptilolite (CPL) was used as reinforcing filler within CS matrix and was acid-activated as previously shown [30]. Briefly, the CPL fraction with sizes in the 0.032–0.050 mm range was activated with 1M HCl aqueous solution at a CPL/solution ratio of 1:10 (*v*/*v*) for 24 h. Afterwards, the CPL particles were washed multiple times with water until no Cl^−^ ions were detected into the washing solutions. The acid-treated CPL particles were dried for 2 h at 105 °C, then at 40 °C for 24 h more, in a vacuum oven. Glutaraldehyde (GA), as aqueous solution with a concentration of 25%, purchased from Sigma–Aldrich, was used as cross-linker. The high-concentrated wastewaters containing Cu^2+^, Zn^2+^, Ni^2+^, Fe^3+^, and Cr^3+^ ions resulted from industrial photoetching processes were provided by Exella Europe SRL, Manufacturing Company of Metal-based Products, located in Cluj–Napoca, Romania.

### 3.2. Methods

CS-based composite cryogel, as a monolith, was prepared in the presence of CPLH^+^ as reinforcing filler, by unidirectional freezing technique. Typically, the CPLH^+^ fraction (0.075 g) with particle sizes in the range of 0.032 and 0.050 mm was dispersed in 5 mL MilliQ water and then was added to 10 g of CS (3 wt.% in 2 wt.% CH_3_COOH solution). This mixture was kept under stirring for 60 min, and then GA solution (0.64 mL, 2.5 wt.%) was added drop–by–drop under vigorous stirring over 30 min. Finally, aliquots from this mixture were transferred into 5 mL syringes, sealed with Parafilm, and then unidirectional frozen in liquid nitrogen, as previously described [49]. The cross-linking reaction was conducted at −18 °C using a CC1-K6 Huber Cryostat for 24 h. Afterwards, the syringes were taken out and maintained at room temperature (RT) for 1 h. After thawing, the cryogels were pushed out of syringes, cut as monoliths of 2.5 cm height, and immersed in 200 mL MilliQ water to wash out the unreacted compounds. Finally, the samples were dried by lyophilization in a Martin Christ, Alpha 1-2LD device (Martin Christ Gefriertrocknungsanlagen GmbH, Osterode am Harz, Germany) for 48 h, at −57 °C and 0.045 mbars. A similar protocol was selected to synthesize, purify, and dry the cross-linked CS cryogel without CPLH^+^. Some characteristics of the samples used as sorbents are summarized in Figure 1. The porosity of cryogels was evaluated by the liquid displacement method [50]. Thus, 0.01 g of dried monoliths were immersed in a known volume of isopropanol (*V_1_*) for 5 min. After the monoliths removal, the volume of isopropanol was measured (*V_3_*). The porosity (*P*, %) was calculated by Equation (4) [50]:

(4) $$P\;\%=\frac{V_{1}-V_{3}}{V_{2}-V_{3}}\times 100$$

where *V_2_* is the total volume of the monoliths impregnated with isopropanol.

The volume fraction of pores in cryogel sorbents (*V_p_*) was also evaluated by Equation (5) [51]:

(5) $$V_{p}=1-\frac{m}{d_{2}V_{T}}$$

where *m* is the weight of dry phase (CS + GA + CPL); *d_2_* is the density of CS (0.6 g cm^−3^); *V_T_* is the total volume of the equilibrium swollen monolith (in water) and was calculated by Equation (6):

(6) $$V_{T}=\pi {\left(\frac{D_{w}}{2}\right)}^{2}l_{w}$$

where *D_w_* is the diameter of the equilibrium swollen monolith; *l_w_* is the length of the equilibrium swollen monolith.

For the CPLH^+^ sorbent, the BET surface area (S_BET_) and the total pore volume were determined using a NOVA 2200e^®^Quantachrome automated gas sorption analyzer (Quantachrome Instruments, Boynton Beach, FL, USA). The NovaWin software version 11.02 (Quantachrome Instruments) was used to process the nitrogen sorption isotherm registered for CPLH^+^ (Figure 1B).

The average pore sizes of cryogel sorbents were estimated using the ImageJ 1.48v analyzing software (National Institutes of Health and the Laboratory for Optical and Computational Instrumentation, University of Wisconsin, USA) [52] from three independent micrographs taken using an environmental scanning electron microscope (Quanta 200, FEI Company, Hillsboro, OR, USA). The water uptake (WU, g g^−1^) was calculated by Equation (7), as previously described [52]:

(7) $$WU=\frac{W_{w}-W_{d}}{W_{d}}$$

where *W_w_* is the weight of swollen samples and *W_d_* is the weight of the dried samples. All experiments were performed in triplicates and their average values were reported in Figure 1A.

### 3.3. Batch Sorption Studies

The sorption performance of all sorbents was performed under competitive conditions, i.e., using a five–component mixture of Cu^2+^, Zn^2+^, Ni^2+^, Fe^3+^, and Cr^3+^ metal ions by batch system. Several sorption experimental parameters, such contact time, pH, sorbent dose, and temperature were investigated to determine their effect on the sorption process. The effect of sorption time was investigated by varying this parameter between 15 and 480 min, while the other parameters were kept constant. For the study of the pH influence, the initial pH value of the HMIs-containing wastewater was adjusted between 1.5 and 5.5 with 0.1 M HCl or 0.1 M NaOH. The effect of sorbent dose on sorption capacity was investigated by varying the amounts of sample into the aqueous mixture of metal ions. The solutions were equilibrated for 6 h at 200 rpm. Sorption experiments were performed at different temperatures to evaluate the temperature effect. The temperature used in this experiment was kept constant at 293, 298, 303, 308, and 313 K. To investigate the sorbent reusability, the metal ions loaded onto CS–based sorbents were eluted with 0.1 M HCl aqueous solution (20 mL) for 6 h. Then, the CS–based sorbents were washed several times with distilled water and were regenerated with 0.1 M NaOH aqueous solution (20 mL) for 6 h. After this treatment, the sorbents were reused in another cycle of sorption.

In each experiment, the supernatants of the solution were filtered using a membrane filter and final concentrations were determined by Flame Atomic Absorption Spectrometry (FAAS) using a high-resolution ContrAA 300 Analytik Jena spectrometer (Analytik Jena, Jena, Germany) equipped with a xenon lamp as a continuum radiation source. During experiment an aspiration rate of about 5 mL min^−1^ was used. Measurements of each analyte were carried out in triplicate. The residual concentration of each metal ion was determined at the characteristic maximum wavelengths of 324 nm for Cu^2+^ ions, 213 nm for Zn^2+^ ions, 232 nm for Ni^2+^ ions, 248 nm for Fe^3+^ ions, and 357 nm for Cr^3+^ ions, respectively.

The amount of metal ion adsorbed at equilibrium (q_e_, mg g^−1^) on all sorbents was calculated as:

(8) $$q_{e}=\frac{\left(C_{0}-C_{e}\right)\times V}{m}$$

*C_o_* – initial metal ion concentration, mg L^−1^; *C_e_* – concentration of the metal ion in aqueous solution at equilibrium, mg L^−1^; *V* – volume of aqueous solution, L; *m* – sorbent dose, g.

The efficiency of metal ion removal (RE, %) from aqueous solution on all sorbents was calculates as:

(9) $$RE\left(\%\right)=\frac{C_{0}-C_{e}}{C_{0}}\times 100$$

*C_o_* and *C_e_* – the same meaning as in Equation (8).

### 3.4. Dynamic Sorption Studies

The five-component solution containing Cu^2+^, Zn^2+^, Ni^2+^, Fe^3+^, and Cr^3+^ metal ions was used in the dynamic sorption procedure. A small glass column calibrated with an internal diameter of 5 cm and length of 16 cm was adapted as the experiment column at RT. The swollen monolith of 2.5 cm height was placed in the column. The top of the column was connected to a Lambda Hiflow peristaltic pump (Lambda Laboratory Instruments, Brno, Czech Republic) to the liquid-processor, and thus providing a continuous flow of five-component solution at 1.33 mL min^−1^. A Lambda OMNICOLL Fraction Collector (Lambda Laboratory Instruments, Brno, Czech Republic) collected effluent samples, which were subsequently analyzed for the residual HMI concentrations, every 15 min. Elution of HMIs was performed by passing of 0.1 M HCl through the fixed-bed column with a flow velocity of 0.42 mL min^−1^.

## 4. Conclusions

The sorption performance of composite cryogels consisting of CS embedding a natural zeolite toward divalent (Cu^2+^, Zn^2+^, and Ni^2+^) and trivalent (Fe^3+^, and Cr^3+^) cations removal from industrial wastewaters was deeply investigated in this work in comparison to those of CS cryogels and of acid-activated zeolite. The effect of several parameters (sorbent dose, contact time, pH, initial metal ion concentration, and temperature) on the sorption properties has been clearly established. The optimum sorption pH for Cu^2+^, Zn^2+^, Ni^2+^, Fe^3+^, and Cr^3+^ cations removal under competitive conditions was 5.0, 5.0, 4.5, 4, and respectively, 3.5. The CSCS_CPLH^+^ composite cryogel exhibited the highest sorption capacity, the maximum theoretical sorption capacities of this composite sorbent according to Sips model toward Cu^2+^, Zn^2+^, Ni^2+^, Fe^3+^, and Cr^3+^ ions were 61.10 mg·g^−1^, 18.67 mg·g^−1^, 12.24 mg·g^−1^, 53.46 mg·g^−1^, and respectively, 0.85 mg·g^−1^. The modeling of kinetics, isotherm, and thermodynamic experimental data indicated a spontaneous chemisorption process for the sorption of all metal cations onto CS–based sorbents. The dynamic sorption studies showed that the CGCS_CPLH^+^ composite sorbent exhibited a high selectivity toward Cu^2+^ ions. The obtained results support the further application of the composite cryogel based on CS and CPLH^+^ for the treatment of industrial wastewaters.

## Figures and Tables

**Table 1:** Kinetic model parameters for the sorption of Cu^2+^, Zn^2+^, and Ni^2+^ ions onto sorbents.

Kinetic Models^a^	$\mathrm{PFO}:\;q_{t}=q_{e}\left(1-exp^{-k_{1}t}\right)$; *k_1_*—Rate Constant of the PFO Kinetic Model, min^−1^				$\mathrm{PSO}:\;q_{t}=\frac{q_{e}^{2}k_{2}t}{1+q_{e}k_{2}t}$; *k_2_*—Rate Constant of the PSO Kinetic Model, g mg^–1^ × min^–1^			
Sorbents					Sorbents			
Metal Ions		CPLH^+^	CGCS	CGCS_ CPLH^+^		CPLH^+^	CGCS	CGCS_ CPLH^+^
**Cu^2+^**	*q_e,calc_*	8.97	35.80	57.15	*q_e,calc_*	8.14	29.42	50.77
	*k_1_*	0.03	0.01	0.02	*k_2_*	0.005	0.0004	0.0005
	*R^2^*	0.99	0.96	0.97	*R^2^*	0.99	0.98	0.98
	*χ^2^*	0.08	5.79	8.71	*χ^2^*	0.08	2.36	5.49
**Zn^2+^**	*q_e,calc_*	0.36	2.84	5.46	*q_e,calc_*	0.32	2.27	4.73
	*k_1_*	0.02	0.01	0.02	*k_2_*	0.09	0.004	0.004
	*R^2^*	0.94	0.96	0.99	*R^2^*	0.98	0.98	0.99
	*χ^2^*	0.0008	0.04	0.03	*χ^2^*	0.0002	0.02	0.01
**Ni^2+^**	*q_e,calc_*	0.16	0.75	1.47	*q_e,calc_*	0.13	0.69	1.34
	*k_1_*	0.01	0.03	0.04	*k_2_*	0.08	0.07	0.03
	*R^2^*	0.94	0.91	0.98	*R^2^*	0.97	0.96	0.99
	*χ^2^*	0.0009	0.005	0.003	*χ^2^*	0.002	0.002	0.001

*^a^q_e,calc_* is the calculated amount of HMIs adsorbed on sorbents, mg·g^−1^.

**Table 2:** Kinetic model parameters for the sorption of Fe^3+^ and Cr^3+^ metal ions onto sorbents.

Kinetic Models		$\mathrm{PFO}:\;q_{t}=q_{e}\left(1-exp^{-k_{1}t}\right)$; *k_1_*—Rate Constant of the PFO Kinetic Model, min^−1^.				$\mathrm{PSO}:\;q_{t}=\frac{q_{e}^{2}k_{2}t}{1+q_{e}k_{2}t}$; *k_2_*—Rate Constant of the PSO Kinetic Model, g mg^–1^ × min^−1^.			
Sorbents						Sorbents			
Metal Ions			CPLH^+^	CGCS	CGCS_ CPLH^+^		CPLH^+^	CGCS	CGCS_ CPLH^+^
**Fe^3+^**	*q_e,calc_*		3.72	24.51	46.63	*q_e,calc_*	2.95	19.68	40.27
	*k_1_*		0.01	0.01	0.02	*k_2_*	0.003	0.0005	0.0004
	*R^2^*		0.96	0.95	0.98	*R^2^*	0.98	0.97	0.99
	*χ^2^*		0.03	1.95	0.57	*χ^2^*	0.06	3.65	4.81
**Cr^3+^**	*q_e,calc_*		0.02	0.05	0.07	*q_e,calc_*	0.02	0.03	0.06
	*k_1_*		0.03	0.01	0.01	*k_2_*	2.08	0.2	0.17
	*R^2^*		0.98	0.94	0.92	*R^2^*	0.99	0.96	0.95
	*χ^2^*		0.0002	0.0006	0.0003	*χ^2^*	0.006	0.0002	0.0005

*^a^q_e,calc_* is the calculated amount of HMIs adsorbed on sorbents, mg g^−1^.

**Table 3:** Thermodynamic parameters for the sorption of Cu^2+^, Zn^2+^, Ni^2+^, Fe^3+^, and Cr^3+^ ions onto investigated sorbents.

Metal Ions	CGCS_CPLH+						
	ΔH°, J mol^−1^	ΔS°, kJ mol^−1^ K^−1^	ΔG°, kJ·mol^−1^				
			293	298	303	308	313
Cu^2+^	74.79	0.318	−18.67	−19.93	−21.34	−23.31	−24.97
Zn^2+^	59.74	0.261	−17.02	−18.16	−19.40	−20.64	−22.34
Ni^2+^	25.22	0.047	−9.42	−10.17	−10.78	−11.32	−11.81
Fe^3+^	86.42	0.356	−18.08	−19.42	−21.27	−23.02	−25.21
Cr^3+^	45.29	0.185	−9.02	−10.34	−11.03	−12.07	−12.80
**CGCS**							
	ΔH°, J·mol^−1^	ΔS°, kJ mol^−1^ K^−1^	ΔG°, kJ·mol^−1^				
			293	298	303	308	313
Cu^2+^	34.42	0.171	−15.61	−16.65	−17.37	−18.33	−19.04
Zn^2+^	83.76	0.329	−12.88	−14.44	−16.03	−17.82	−19.45
Ni^2+^	46.86	0.176	−4.89	−5.74	−6.78	−7.67	−8.34
Fe^3+^	37.31	0.476	−14.24	−15.19	−16.10	−16.90	−17.80
Cr^3+^	104.93	0.188	−7.83	−9.09	−10.03	−10.91	−11.61
**CPLH^+^**							
	ΔH°, J·mol^−1^	ΔS°, kJ mol^−1^ K^−1^	ΔG°, kJ·mol^−1^				
			293	298	303	308	313
Cu^2+^	14.38	0.090	−12.12	−12.55	−13.04	−13.46	−13.92
Zn^2+^	26.17	0.114	−7.16	−7.83	−8.37	−8.96	−9.45
Ni^2+^	19.61	0.077	−3.02	−3.45	−3.95	−4.26	−4.55
Fe^3+^	17.03	0.093	−10.43	−10.95	−11.43	−11.88	−12.30
Cr^3+^	60.08	0.223	−5.21	−6.71	−7.83	−8.75	−9.76

## Supplementary Materials

The following are available online, Figure S1: Sorption isotherms of Cu^2+^ ions at pH = 5 (a), Zn^2+^ ions at pH = 5 (b), Ni^2+^ ions at pH = 4.5 (c), Fe^3+^ ions at pH = 4 (d), and Cr^3+^ ions at pH = 3.5 (e) onto CPLH^+^, CGCS, and CGCS_CPLH^+^ sorbents (sorbent dose = 3.5 g L^−1^, V = 10 mL, T = 293 K, N = 125 rpm, t = 24 h, C_0_ = 50−1000 mg/L), Table S1: Values of the parameters for the fitted isotherm models onto CPLH^+^, Table S2: Values of the parameters for the fitted isotherm models onto CGCS, Table S3: Values of the parameters for the fitted isotherm models onto CGCS_CPLH^+^, Table S4: Comparison of the q_m_ values of various sorbents obtained according to the best fitted isotherm model onto HMIs sorption data.
