# Supplementary material for: A Comparative Study on Cu2+, Zn2+, Ni2+, Fe3+, and Cr3+ Metal Ions Removal from Industrial Wastewaters by Chitosan-Based Composite Cryogels
Source: Molecules. 2020 Jun 8;25(11):2664. doi: 10.3390/molecules25112664 (PMC7321311; doi:10.3390/molecules25112664)
Supplement: Supplementary file 1 [file molecules-25-02664-s001.pdf]

Supplementary Information

A Comparative Study on Cu^{2+}, Zn^{2+}, Ni^{2+}, Fe^{3+}, and Cr^{3+} Metal Ions Removal from Industrial Wastewaters by Chitosan-Based Composite Cryogels

D. Humelnicu,[1] E.S. Dragan,[2] M. Ignat,[1,3] M.V. Dinu[2*]

[1]"Alexandru Ioan Cuza" University of Iasi, Faculty of Chemistry, Bd. 11 Carol I, 700506 Iasi, Romania
[2]"Petru Poni" Institute of Macromolecular Chemistry, Department of Functional Polymers, Grigore Ghica Voda Alley 41A, Iasi 700487, Romania
[3]Laboratory of Inorganic Polymers, "Petru Poni" Institute of Macromolecular Chemistry, Grigore Ghica Voda Alley 41A, 700487 Iasi, Romania
*corresponding author: *vdinu@icmpp.ro*

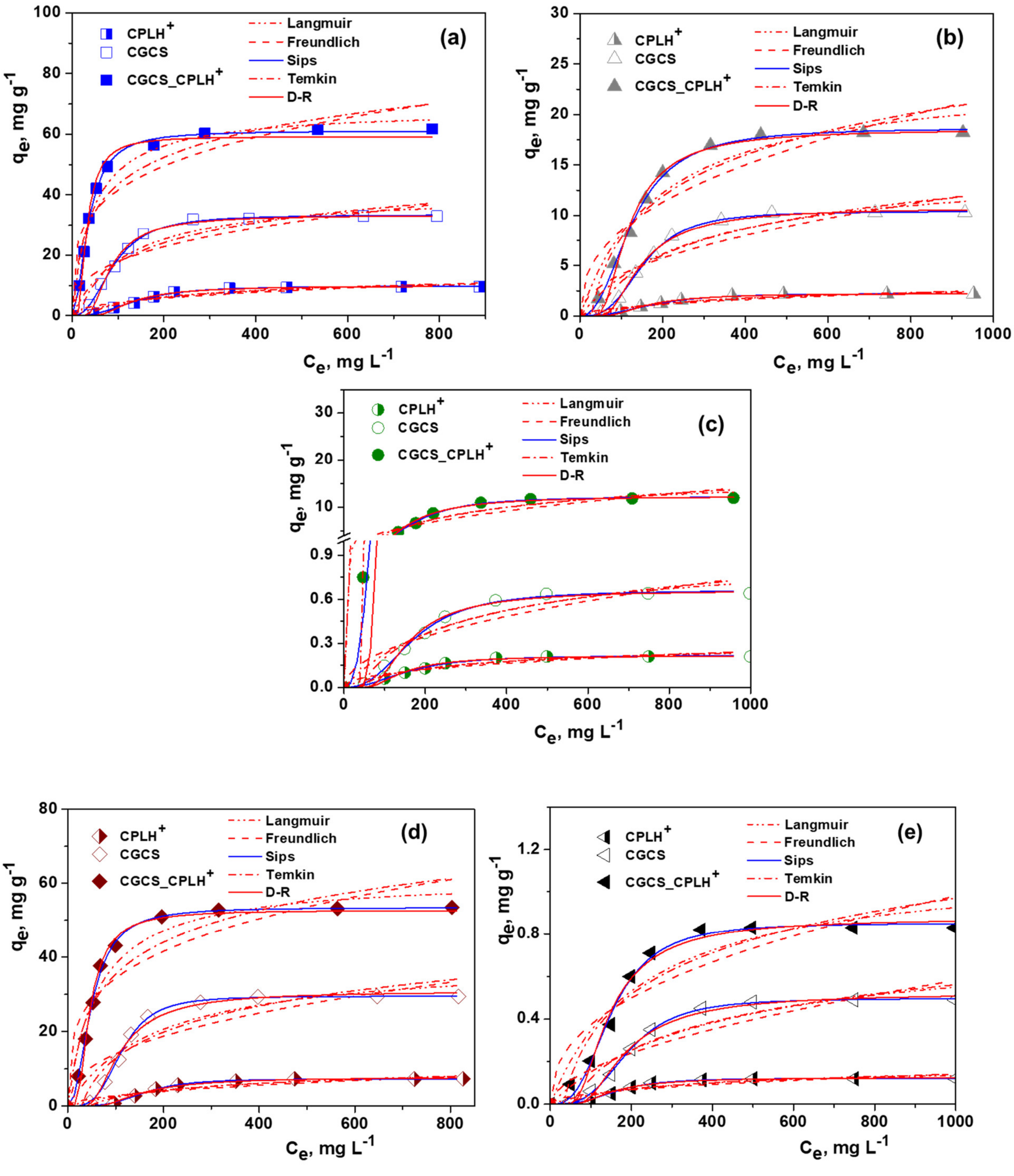

Figure S1. Sorption isotherms of Cu^{2+} ions at pH = 5 (a), Zn^{2+} ions at pH = 5 (b), Ni^{2+} ions at pH = 4.5 (c), Fe^{3+} ions at pH = 4 (d), and Cr^{3+} ions at pH = 3.5 (e) onto $CPLH^+$, CGCS, and $CGCS\_CPLH^+$ sorbents (sorbent dose = 3.5 g L^{-1}, V = 10 mL, T = 293 K, N = 125 rpm, t = 24 h, C_0 = 50–1000 mg/L).

Isotherm Sorption Models

The distribution of the adsorbate species between liquid and adsorbent is described by mathematical models (Figure S1). Usually, Langmuir or Freundlich model describe accurately the system in the case of two parameters [43]. Langmuir model assumes monolayer adsorption onto homogenous surface where the binding sites have equal affinity and energy, and there is no transmigration or interaction between the molecules. Hence, it can reach saturation (Eq. (1)) [24,41-

43]. The Freundlich model assumes multilayer adsorption on heterogeneous surface and the amount of adsorbed adsorbate increases infinitely with an increase in concentration (Eq. (2)) [24,41-43].

$$q_e = \frac{q_m K_L C_e}{1+K_L C_e} \quad (3)$$

$$q_e = K_F C_e^{1/n} \quad (4)$$

where q_e (mg g^{-1}) is the equilibrium concentration in the solid phase; q_m (mg g^{-1}) is the maximum theoretical sorption capacity; K_L (L mg^{-1}) is the Langmuir constant; K_F (mg $g^{-1}mg^{-1/n}L^{-1/n}$) is the Freundlich constant, and n is the constant depicting both the nature and strength of sorption process, and of active sites distribution, related to the surface heterogeneity. Regarding the significance of n; sorption is linear if n = 1, chemical process if n > 1, and physical process if n < 1 [24].
The separation factor from the Langmuir isotherm equation is given as follows:

$$R_L = \frac{1}{1+K_L C_0} \quad (5)$$

Sips (Eq. (6)) isotherm model is also applied to obtain meaningful results on the HMIs sorption mechanism (Figure S1). Its constants and correlation coefficient in single system are presented to Tables S1-S3 for all studied sorbents.

$$q_e = \frac{q_m a_S C_e^N}{1+a_S C_e^N} \quad (6)$$

a_S is the Sips constant, while the other parameters have the same meaning like in Eqs. (3) and (4).
Temkin model is also tested (Eq. (7)) because it takes into account the heterogeneity of the surface

[24,41-43]:

$$q_e = \frac{RT}{b_T}\ln{(a_T C_e)} \text{ and } E_T = \frac{RT}{b_T} \quad (7)$$

b_T is Temkin constant and a_T (L g^{-1}) is the equilibrium binding constant while E_T (kJ mol^{-1}) is related to the heat of adsorption. The positive values of the heat of sorption (0.071< E_T < 56.34 kJ mol^{-1}) (Tables S1-S3) revealed that the process was endothermic [24].

The suitability of Dubinin-Radushkevich (D-R) model was also checked to distinguish physical and chemical sorption through the mean free energy for heterogeneous surfaces (Eq. (9)) [24,36,43]. The mean free energy of sorption, E (kJ mol^{-1}), defined as the free energy of one mole of ions that is transferred from the infinity of solution to the surface of a solid, calculated with Eq. (10), is typically used to estimate the sorption type [36].

$$q_e = q_{DR} e^{-K_{DR}\varepsilon^2} \text{ and } \varepsilon = RTln\left(1+\frac{1}{C_e}\right) \quad (9)$$

$$E = \frac{1}{(2K_{DR})^{1/2}} \quad (10)$$

q_{DR} (mg g^{-1}) is the maximum sorption capacity; K_{DR} (mol^2 kJ^{-2}) is D–R isotherm constant; R (J $mol^{-1}K^{-1}$) is the gas constant; T is the temperature in Kelvin. Values of E lower than 8 kJ mol^{-1} depict a physical sorption, while E values ranging from 8 to 16 kJ mol-1 characterize a sorption process occurring by an ion exchange mechanism. E values higher than 40 kJ mol^{-1} indicate chemisorption as the mechanism of sorption [24,36,43].

Table S1. Values of the parameters for the fitted isotherm models onto $CPLH^{+}$.

Isotherm Parameters	Metal ions Cu^{2+}	Zn^{2+}	Ni^{2+}	Fe^{3+}	Cr^{3+}
**Langmuir**					
R^2	0.93	0.93	0.94	0.90	0.91
q_m	12.85	3.19	0.29	10.78	0.17
K_L	0.005	0.003	0.004	0.003	0.004
R_L	0.46	0.84	0.52	0.59	0.97
**Freundlich**					
R^2	0.84	0.86	0.87	0.82	0.83
n	2.39	2.02	2.29	2.04	2.17
K_F	0.63	0.084	0.012	0.30	0.006
**Sips**					
R^2	0.99	0.99	0.99	0.99	0.99
q_m	9.76	2.24	0.22	7.34	0.12
N	2.57	2.57	2.27	3.59	3.05
a_S	2.93×10^{-6}	1.74×10^{-6}	9.89×10^{-6}	1.18×10^{-8}	1.1×10^{-7}
**Temkin**					
R^2	0.93	0.95	0.95	0.92	0.92
a_T	0.033	0.023	0.03	0.023	0.025
b_T	0.77	3.07	34.20	0.88	56.34
E_T	3.17	0.792	0.0711	2.74	0.043
**D-R**					
R^2	0.98	0.99	0.98	0.99	0.99
q_{DR}	9.82	2.24	0.216	7.75	0.126
E	15.15	12.5	13.69	12.50	12.98
K_{DR}	2.2×10^{-3}	3.3×10^{-3}	2.7×10^{-3}	3.16×10^{-3}	3×10^{-3}

Table S2. Values of the parameters for the fitted isotherm models onto CGCS.

Isotherm Parameters	Metal ions Cu^{2+}	Zn^{2+}	Ni^{2+}	Fe^{3+}	Cr^{3+}
**Langmuir**					
R^2	0.93	0.91	0.93	0.89	0.91
q_m	41.16	14.84	0.93	39.64	0.77
K_L	0.008	0.004	0.003	0.005	0.002
R_L	0.35	0.80	0.59	0.46	0.98
**Freundlich**					
R^2	0.83	0.83	0.86	0.80	0.84
n	2.28	2.09	2.07	2.43	1.81
K_F	3.63	0.46	0.026	2.12	0.013
**Sips**					
R^2	0.99	0.99	0.99	0.99	0.99
q_m	33.42	10.40	0.66	29.49	0.49
N	2.46	3.09	2.56	3.58	3.3
a_S	2×10^{-6}	1.67×10^{-7}	1.86×10^{-6}	4.4×10^{-8}	2.82×10^{-8}
**Temkin**					
R^2	0.91	0.93	0.95	0.89	0.93
a_T	0.062	0.025	0.024	0.035	0.019
b_T	0.253	0.641	10.473	0.240	12.821
E_T	9.59	3.79	0.232	10.11	0.189
**D-R**					
R^2	0.98	0.99	0.99	0.99	0.99
q_{DR}	33.31	10.75	0.664	30.79	0.521
E	23.81	13.51	12.37	18.52	10.64
K_{DR}	9×10^{-4}	2.8×10^{-3}	3.27×10^{-3}	1.5×10^{-3}	4.46×10^{-3}

Table S3. Values of the parameters for the fitted isotherm models onto $CGCS\_CPLH^{+}$.

Isotherm Parameters	Metal ions Cu^{2+}	Zn^{2+}	Ni^{2+}	Fe^{3+}	Cr^{3+}
**Langmuir**					

R^2	0.96	0.94	0.93	0.95	0.92
q_m	68.28	24.17	17.18	61.54	1.14
K_L	0.023	0.005	0.004	0.017	0.004
R_L	0.16	0.76	0.52	0.20	0.97
**Freundlich**					
R^2	0.86	0.86	0.86	0.84	0.83
n	4.00	2.44	2.08	3.70	2.38
K_F	12.91	1.3	0.53	9.66	0.054
**Sips**					
R^2	0.99	0.99	0.99	0.99	0.99
q_m	61.10	18.67	12.24	53.46	0.85
N	1.91	2.37	2.64	2.21	2.90
a_S	0.001	10^{-6}	1.52×10^{-6}	1.8×10^{-5}	4.58×10^{-7}
**Temkin**					
R^2	0.92	0.94	0.94	0.90	0.91
a_T	0.295	0.04	0.026	0.173	0.031
b_T	0.189	0.412	0.571	0.196	8.644
E_T	12.84	5.89	4.26	12.42	0.282
**D-R**					
R^2	0.98	0.98	0.99	0.98	0.98
q_{DR}	59.27	18.53	12.34	52.72	0.874
E	62.50	15.87	13.51	43.47	13.88
K_{DR}	1.3×10^{-4}	2×10^{-3}	2.8×10^{-3}	2.6×10^{-4}	2.63×10^{-3}

Table S4. Comparison of the q_m values of various sorbents obtained according to the best fitted isotherm model onto HMIs sorption data .

Sorbents	pH	Isotherm	Metal ions	q_m, mg g^{-1}	Refs.
NaCPL from Romania	4	Langmuir	Cu^{2+}	12.16	[6]
			Zn^{2+}	7.32	
			Ni^{2+}	3.30	
MnO2-coated zeolite from Iran	5.5	Langmuir	Ni^{2+}	10.51	[7]
NaCPL from Serbia		Sips	Ni^{2+}	16.64	[8]
CPL from Ukraine	7.5	Langmuir	Cu^{2+}	25.69	[9]
			Ni^{2+}	15.55	
Natural aluminosilicates modified by N,N'-bis(3-triethoxysilylpropyl)thiocarbamide		Langmuir	Cu^{2+}	29.95	[45]
			Ni^{2+}	80.00	
			Zn^{2+}	2.83	
CPLH+ from Romania	5	Sips	Cu^{2+}	9.76	This study
	5		Zn^{2+}	2.24	
	4.5		Ni^{2+}	0.22	
	4		Fe^{3+}	7.34	
	3.5		Cr^{3+}	0.12	
CS flakes	3.8	Langmuir	Cr^{3+}	138.04	[12]
Diacetylmonoxine modified CS	5	Langmuir	Ni^{2+}	135.00	[17]
Glutamic-CS hydrogel	5	Langmuir	Cu^{2+}	83.33	[20]
	5		Ni^{2+}	103.4	
CS gel	5	Langmuir	Cu^{2+}	75.4	[21]
CS	3	Langmuir	Fe^{3+}	90.09	[42]
CS-ECH				72.46	
CS-GLA				51.55	
CS-EDGE				46.30	
CGCS	5	Sips	Cu^{2+}	33.42	This study
	5		Zn^{2+}	10.40	
	4.5		Ni^{2+}	0.66	
	4		Fe^{3+}	29.49	
	3.5		Cr^{3+}	0.49	
CS/starches-g-PAN cryobeads	5	Langmuir	Cu^{2+}	100.6	[23]
	6		Ni^{2+}	83.25	
CS/poly(vinyl alcohol) beads	5	Langmuir	Cu^{2+}	38.68	[24]
CS/poly(vinyl amine) composite beads	4.5	Langmuir	Ni^{2+}	143.73	[38]
			Cr^{3+}	153.35	
CS/PAAm/CPL monoliths	4.5	Langmuir	Cu^{2+}	219.59	[36]
CGCS_CPLH+	5	Sips	Cu^{2+}	61.10	This study
	5		Zn^{2+}	18.67	

	4.5	Ni^{2+}	12.24
	4	Fe^{3+}	53.46
	3.5	Cr^{3+}	0.85

The references from *Supplementary Information* correspond to those presented in the main text of the manuscript.
